# Perspectives of healthcare professionals on training for quantitative G6PD testing during implementation of tafenoquine in Brazil (QualiTRuST Study)

**DOI:** 10.1371/journal.pntd.0012197

**Published:** 2024-06-05

**Authors:** Alicia Santos, Marcelo Brito, Evellyn Silva, Felipe Rocha, Ana Oliveira, Rafaela Dávila, Hiran Gama, Jéssica Albuquerque, Mena Paiva, Djane Baía-Silva, Vanderson Sampaio, Patrícia Balieiro, Rosilene Rufatto, Penny Grewal Daumerie, Cássio Peterka, Francisco Edilson Lima, Wuelton Monteiro, Ana Arcanjo, Ricardo Silva, Dhelio Batista Pereira, Marcus Lacerda, Felipe Murta

**Affiliations:** 1 Fundação de Medicina Tropical Dr Heitor Vieira Dourado, Manaus, Brazil; 2 Universidade do Estado do Amazonas, Manaus, Brazil; 3 Centro de Pesquisa em Medicina Tropical de Rondônia (CEPEM), Porto Velho, Brazil; 4 Medicines for Malaria Venture, Geneva, Switzerland; 5 Brazilian Ministry of Health, Brasília, Brazil; 6 Fundação de Vigilância em Saúde, Manaus, Brazil; 7 Instituto Leônidas & Maria Deane, Fiocruz, Manaus, Brazil; 8 University of Texas Medical Branch, Galveston, Texas, United States of America; Menzies School of Health Research, AUSTRALIA

## Abstract

Effective radical cure of *Plasmodium vivax* malaria is essential for malaria elimination in Brazil. *P*. *vivax* radical cure requires administration of a schizonticide, such as chloroquine, plus an 8-aminoquinoline. However, 8-aminoquinolines cause hemolysis in individuals with glucose-6-phosphate dehydrogenase (G6PD) deficiency, requiring prior screening to exclude those at risk. Brazil is pioneering the implementation of tafenoquine, a single-dose 8-aminoquinoline indicated for *P*. *vivax* patients with >70% of normal G6PD activity. Tafenoquine implementation in Manaus and Porto Velho, two municipalities located in the western Brazilian Amazon, included comprehensive training of healthcare professionals (HCPs) on point-of-care quantitative G6PD testing and a new treatment algorithm for *P*. *vivax* radical cure incorporating tafenoquine. Training was initially provided to higher-level facilities (phase one) and later adapted for primary care units (phase two). This study analyzed HCP experiences during training and implementation and identified barriers and facilitators. In-depth interviews and focus discussion groups were conducted 30 days after each training for a purposive random sample of 115 HCPs. Thematic analysis was employed using MAXQDA software, analyzing data through inductive and deductive coding. Analysis showed that following the initial training for higher-level facilities, some HCPs did not feel confident performing quantitative G6PD testing and prescribing the tafenoquine regimen. Modifications to the training in phase two resulted in an improvement in understanding the implementation process of the G6PD test and tafenoquine, as well as in the knowledge acquired by HCPs. Additionally, knowledge gaps were addressed through *in situ* training, peer communication via a messaging app, and educational materials. Training supported effective deployment of the new tools in Manaus and Porto Velho and increased awareness of the need for pharmacovigilance. A training approach for nationwide implementation of these tools was devised. Implementing quantitative G6PD testing and tafenoquine represents a significant shift in *P*. *vivax* malaria case management. Consistent engagement with HCPs is needed to overcome challenges in fully integrating these tools within the Brazilian health system.

## Author summary

Brazil has implemented tafenoquine and quantitative G6PD testing as a crucial component of its comprehensive strategy to eliminate *Plasmodium vivax* malaria. Training of healthcare professionals (HCPs) has played a pivotal role in facilitating the adoption of these new strategies. In any implementation, it is necessary to identify barriers and facilitators and consider data that cannot typically be collected in purely quantitative studies. This qualitative study describes the perceptions of HCPs regarding the training they received and the knowledge gaps that only became apparent in their professional practice after the training. These insights were instrumental in developing various communication and continuing education strategies that enhanced HCP knowledge following training. Finally, the study presents a training approach for applying quantitative G6PD testing and the tafenoquine treatment regimen, including the most effective educational activities and materials to support the HCP learning process.

## Introduction

*Plasmodium vivax* malaria is a neglected tropical disease that is widespread in developing countries and lacks attention, funding, and resources relative to *P*. *falciparum* malaria [1]. Nevertheless, *P*. *vivax* malaria can cause severe symptoms and complications, with a substantial impact on the health and economic wellbeing of affected communities [2,3].

Around 43 million people are at risk of malaria in Brazil [4]. The country has targeted malaria elimination by 2030 as a key element of the United Nations Sustainable Development Goals [5]. *P*. *vivax* is the dominant parasite in the country, causing 83% of cases in 2021 [4]. The Amazon region has the most *P*. *vivax* malaria cases and is the main source of imported cases in extra-Amazon regions [6]. *P*. *vivax* elimination poses significant technical challenges [7]. Unlike *P*. *falciparum*, *P*. *vivax* has a dormant liver stage (hypnozoite), carried silently until reactivation, causing relapses weeks or months following the initial infection. Relapses increase the disease burden, promote transmission, and allow the parasite to evade malaria control activities. Hypnozoites are insensitive to antimalarials used for acute infection, and radical cure requires a schizonticide to clear the blood-stage parasites and an 8-aminoquinoline to target hypnozoites [8].

Until recently, primaquine was the only 8-aminoquinoline available for *P*. *vivax* relapse prevention. However, adherence to a 7- or 14-day primaquine dosing regimen is challenging, and the effectiveness of unsupervised therapy is limited [9–11]. Additionally, cytochrome P450 2D6 (CYP2D6) polymorphisms that impair primaquine conversion to its active metabolite are associated with an increased recurrence risk [12].

*P*. *vivax* elimination requires new and innovative strategies. In 2019, Brazil became the first malaria endemic country to approve the 8-aminoquinoline tafenoquine for *P*. *vivax* radical cure [13,14]. Tafenoquine is administered as a single 300 mg dose, and efficacy does not appear to be dependent on CYP2D6 activity [15,16]. In a randomized placebo-controlled trial, a combination of chloroquine and tafenoquine reduced recurrence risk by 70% versus chloroquine monotherapy after 6 months [16]. In a double-blind randomized controlled trial, recurrence-free efficacy at 6 months was similar for tafenoquine and 14-day primaquine [17].

Individuals with glucose-6-phosphate dehydrogenase (G6PD) deficiency are at risk of acute hemolytic anemia (AHA) following primaquine or tafenoquine [18–20]. AHA is a severe complication, usually requiring hospitalization, and can be avoided if G6PD status is known. G6PD deficiency prevalence is around 6% in Brazil, and prior G6PD testing is required before administration of 8-aminoquinolines to exclude those at risk [21,22]. To support *P*. *vivax* radical cure implementation, a novel point-of-care quantitative G6PD diagnostic was operationally evaluated in the Brazilian Amazon and found to be cost-effective and feasible with good acceptability [23,24].

In 2021, the Brazilian Ministry of Health updated the *P*. *vivax* malaria treatment guidelines in the cities of Manaus (Amazonas State) and Porto Velho (Rondônia State) to recommended point-of-care quantitative G6PD testing, followed by chloroquine and an 8-aminoquinoline: single-dose tafenoquine (300 mg) for G6PD-normal patients ≥16 years old (not pregnant or breastfeeding), 7-day primaquine (0.5 mg/kg/day) for G6PD-normal or G6PD-intermediate patients ≥6 months old (not pregnant or breastfeeding or breastfeeding >1 month), and once-weekly primaquine (0.75 mg/kg/week) for 8 weeks for G6PD-deficient patients ≥6 months old (not pregnant or breastfeeding or breastfeeding >1 month) [25–27]. Pregnant women, women breastfeeding for ≤1 month and children <6 months received chloroquine only [25].

This new treatment algorithm was a major change to *P*. *vivax* malaria case management. Introducing a new process or tool requires healthcare professionals (HCPs) to be trained on its use, and then integrate the novelty into existing routines and workflows [28,29]. This can be challenging, especially if the new technique requires additional steps or unfamiliar equipment. Understanding HCP perceptions and experiences during implementation provides valuable insights into training effectiveness to identify potential barriers and facilitators [30,31]. This understanding is critical in malaria elimination, where communication gaps can undermine community acceptance of novel approaches [30,31].

The Tafenoquine Roll-out Study (TRuST) was a real-world observational study assessing the feasibility of appropriate *P*. *vivax* radical cure in Manaus and Porto Velho municipalities before national rollout. The study demonstrated that the new treatment algorithm was highly feasible in the Brazilian Amazon, with acceptable safety and high adherence to the protocol for quantitative G6PD testing prior to tafenoquine [32]. This companion qualitative study (QualiTRuST) considers HCP experiences during the TRuST protocol implementation. It aims to understand HCP perceptions of training for quantitative G6PD testing and tafenoquine use. The objective was to identify barriers and recognize facilitators to support effective national deployment of the new *P*. *vivax* treatment algorithm.

## Methods

### Ethics statement

The Ethics Review Board at the Fundação de Medicina Tropical Dr Heitor Vieira Dourado in Manaus and the National Research Ethics Committee (CONEP) in Brasília, Brazil (CAAE: 47598921.2.0000.0005) approved the protocol for both study sites. Eligible HCPs provided their signed consent prior to participation. The study followed the COREQ (Consolidated Criteria for Reporting Qualitative Research) guidelines to ensure high-quality reporting of the research process and findings [33] (S1 File).

### Study context

With the aim of reducing *P*. *vivax* malaria recurrence, improving patient adherence and ensuring patient safety, in 2021 the Brazilian Unified Health System implemented a revised treatment algorithm for *P*. *vivax* radical cure in two municipalities. This included quantitative G6PD testing using the STANDARD G6PD test (SD Biosensor, Suwon-si, Republic of Korea) and the single-dose 8-aminoquinoline tafenoquine (GSK, Ware, UK) for eligible patients based on age and G6PD status. The STANDARD G6PD test is a handheld, battery operated, portable analyzer and requires a 10 μL capillary blood sample collected with a pipette sample collector (STANDARD Ezi Tube+, SD Biosensor, Suwon-si, Republic of Korea), reagents (buffer) and a test strip which is inserted into the analyzer. The test provides G6PD enzyme activity as a ratio to hemoglobin (U/g Hb), as well as a hemoglobin level (g/dL). The manufacturer defines G6PD status as deficient (≤4.0 U/g Hb), intermediate (4.1–6.0 U/g Hb), or normal (≥6.1 U/g Hb). To use the device, the healthcare provider must collect a sufficient amount of blood using the pipette, transfer the sample to the provided sample tube with buffer and mix the buffer eight to ten times. Then using a new pipette, the mixed sample must be applied to the test strip which is inserted into the analyzer, the analyzer cover is then quickly closed and after around 2 minutes the results are read on the digital display screen and then interpreted. Quantitative G6PD testing is essential for the safe administration of 8-aminoquinolines; ensuring that only those with G6PD enzyme activity exceeding 70% of normal receive tafenoquine and only those with more than 30% of normal G6PD activity receive daily primaquine. Thus, it was key that both new innovations were deployed concurrently and with high adherence and acceptability for HCPs and patients. To gain insights into how this could best be achieved, the treatment algorithm was initially implemented in Manaus and Porto Velho. The first phase occurred in higher-level health units, while the second phase, starting three months later, took place in lower-level health units. In this context, the QualiTRusT study was conducted to provide quick qualitative information for potential adaptations and improvements in the implementation process.

### Study design

#### Sampling

This study employed rapid qualitative research methods [34], in a purposive random sample of trained HCPs [35], to provide timely insights in their training experiences focusing on quantitative G6PD testing and tafenoquine use. The purposive sample size was based on the principle of saturation, where in-depth individual interviews (IDIs) and focus group discussions (FGDs) are performed until a clear pattern appears and subsequent interviews/groups do not produce novel information [35].

Eligible participants worked daily with *P*. *vivax* malaria diagnosis, treatment, and health education, or oversaw a facility treating malaria patients, had received the training, and consented to participation. Sampling was distributed equally between Manaus and Porto Velho, with health facilities selected at random and participants then randomly selected to minimize possible health manager influence on participant choice. HCPs who met the study eligibility criteria were invited to participate in the research.

#### Study sites and health facilities

Training was conducted in two phases aligned to the TRuST implementation plan [14]. In the first phase, training, G6PD tests, and tafenoquine were delivered to higher-level health facilities (referral hospitals, general hospitals, and emergency care units). The second phase extended training to lower-level health facilities (basic health units, basic family health units, and primary care facilities). Overall, training sessions were provided to 659 HCPs from 43 centers throughout the implementation process in Manaus (Amazonas State) and Porto Velho (Rondônia State) municipalities in western Brazilian Amazonia, from 12th June 2021 to 6th June 2022.

**Fig 1:**
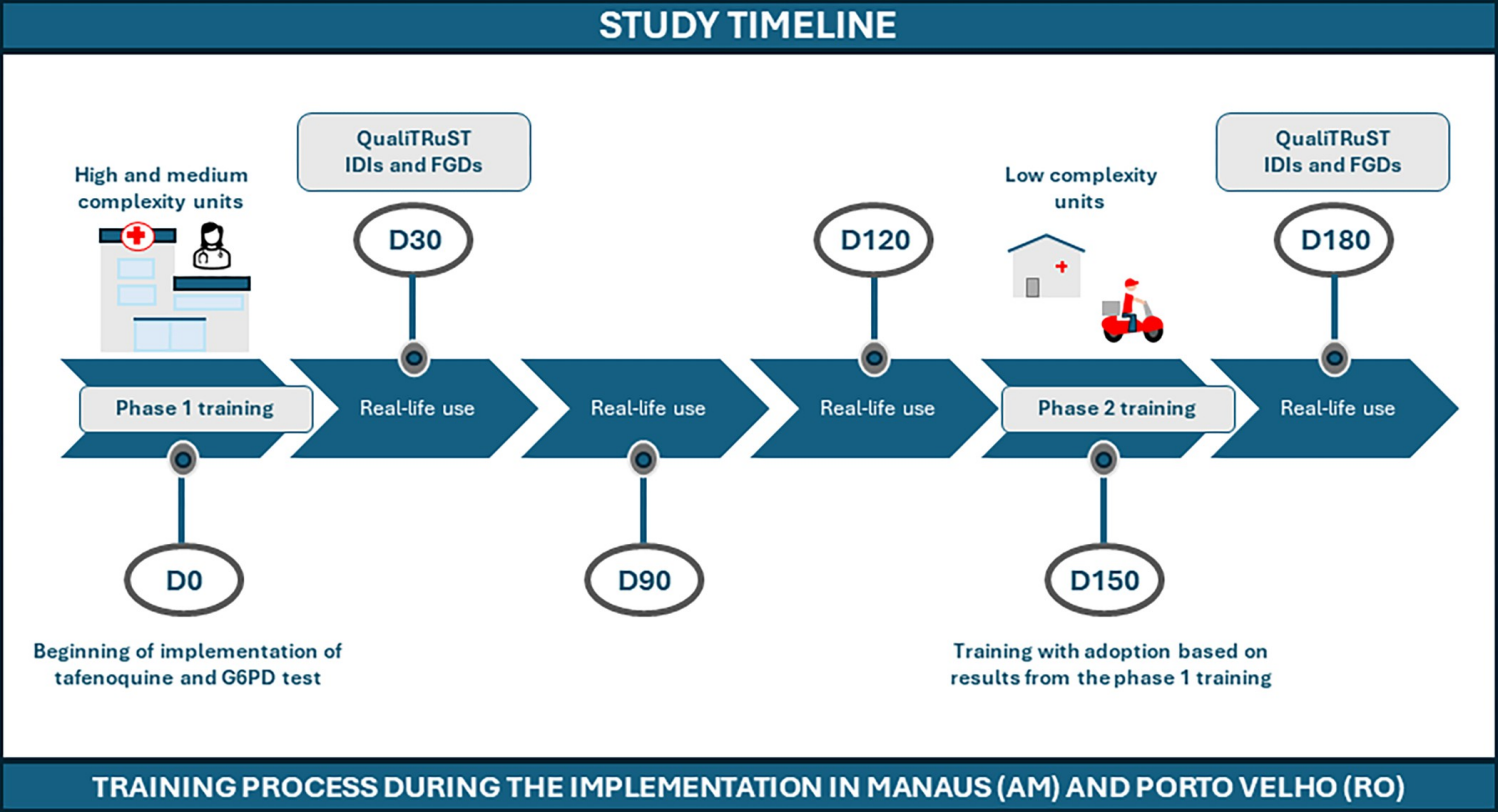
Timeline of training and qualitative data collection periods during the implementation process. F. Murta, created in Canva.

To assess HCP training impact and identify immediate gaps for knowledge enhancement, IDIs and FGDs were conducted 30 days after each training phase was completed (Fig 1). The research team considered this period optimal because it was long enough to allow HCPs to formulate their impressions as they adopted the new procedures within their daily routines, while allowing the team to swiftly identify potential implementation gaps that could affect the professionals’ routines and the implementation of quantitative G6PD testing and tafenoquine. Qualitative analysis findings from the first training phase were incorporated into the second training phase delivered around five months later, with further iterations incorporated as the second phase progressed (Fig 1 and S1 File).

### HCP training process

**Fig 2:**
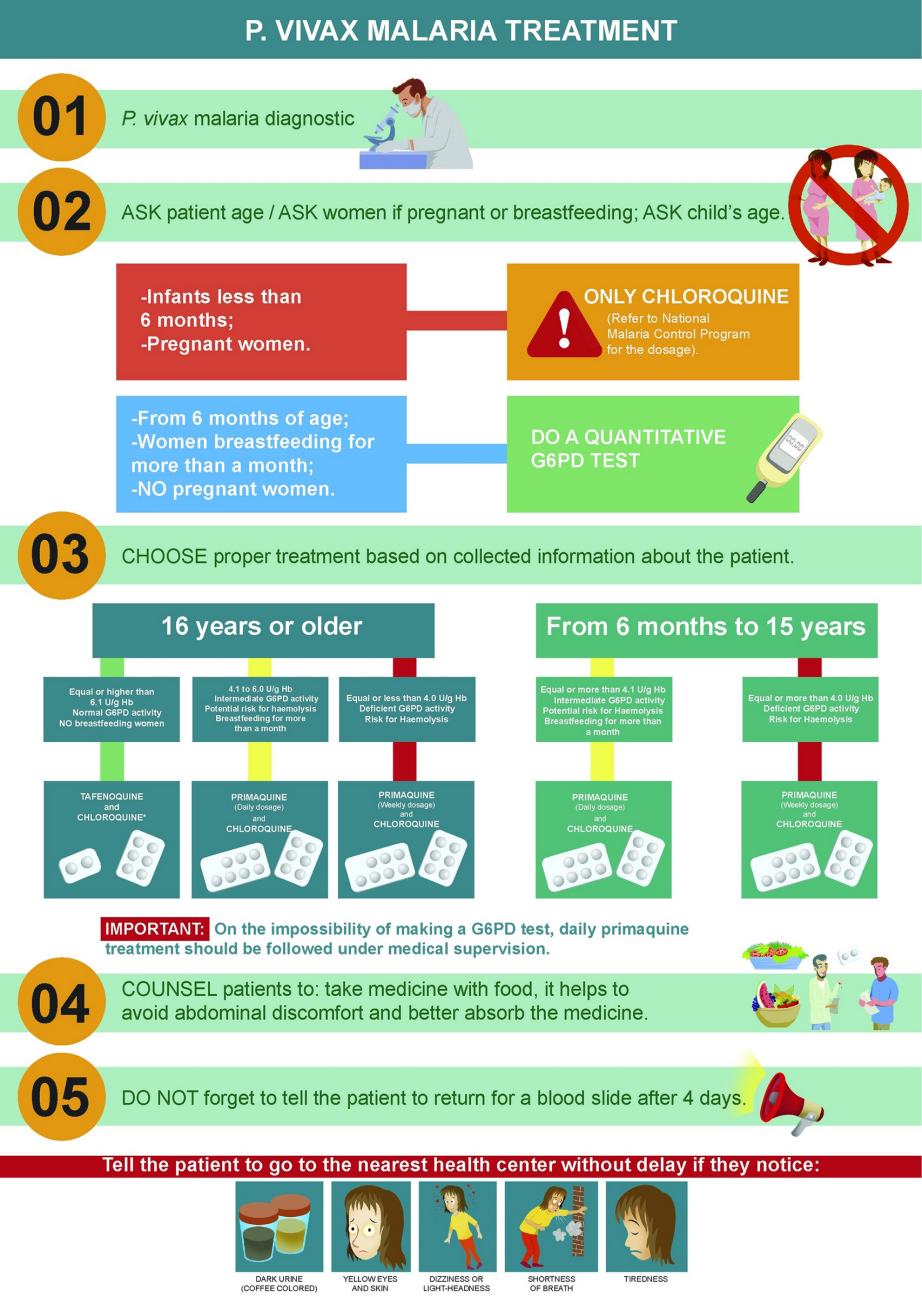
New treatment algorithm adopted in Manaus and Porto Velho municipalities during the implementation of quantitative G6PD testing and tafenoquine.

Training was organized by the Ministry of Health and local governments and supported by the TRuST study [32]. Training was divided into four modules to promote the logical assimilation of information covering: (a) the new treatment algorithm for *P*. *vivax* radical cure (Fig 2); (b) G6PD deficiency and hemolysis signs; (c) G6PD diagnosis; and (d) the updated version of the malaria surveillance form for the Brazilian epidemiological database (SIVEP-Malária).

The first training phase, including HCPs from higher-level health facilities, took place in the three months prior to the first implementation phase (September 2021). However, in Porto Velho there was an interruption in quantitative G6PD test supplies at higher level facilities in mid-October, during which time implementation was suspended, with deployment resuming in mid-November. The second training phase, including HCPs from lower-level facilities was delivered during the month preceding the second implementation phase which started in February 2022. HCP training had different content and delivery for physicians and non-physician HCPs, as physicians had a higher level of pre-existing knowledge and training.

In both phase one and phase two, physician training was delivered via an online platform created using Moodle, an open-source learning management system. This approach was taken to minimize disruptions to working practices amid COVID-19 restrictions. The training assumed detailed clinical knowledge of malaria and focused on the new treatment algorithm, divided into three 15-minute videos. After completing the training, physicians completed a test, with a minimum passing grade of 70% (S2 File).

Non-physician HCP training was more extensive and followed pedagogical approaches used by the Ministry of Health and local governments with in-person theoretical and practical classes on the same day. The same training team conducted both training phases, consisting of two professionals with expertise in quantitative G6PD testing and the new treatment algorithm plus three other professionals (biochemists and pharmacists) who supported and mentored the HCPs undergoing training.

In phase one, the non-physician HCPs received a single 8-hour training session divided into two parts: 4 hours of theoretical instruction and 4 hours of practical G6PD testing activities. In phase two, although still completed in one day, theoretical classes were structured into 15-minute sessions, alternating with practical activities related to the subject matter. This approach provided all non-physician HCPs with frequent opportunities to apply their knowledge and engage in hands-on learning (S3 File). The same slide set was used for the theoretical class in both training phases. The slides were built on experience gained in a previous study evaluating quantitative G6PD testing in Brazil [23]. At the conclusion of each training session, an independent evaluator assessed HCPs on their theoretical knowledge and practical skills, with a score of at least 70% needed to pass. If any HCP did not attain proficiency, they were scheduled for retraining within 15 days. Following training completion, HCPs were provided with educational materials in paper and electronic formats to support application of the knowledge acquired during the training (Table 1).

**Table 1:** Printed materials used in both trainings*.

Type	Title	Brief description
Job aid	Guidelines for the Use of the G6PD Activity Test	Key insights into the G6PD test and its role in guiding antimalarial treatment. The test procedure is detailed succinctly, accompanied by instructions for proper storage and transportation to ensure accurate results
Poster	Safety in Tafenoquine Prescription	Material emphasizing the steps required when dispensing tafenoquine, as the drug cannot be dispensed without quantitative G6PD testing
Job aid	Guidelines for Filling Out SIVEP-Malária	Specific guidelines to assist in completing the SIVEP-Malária form, specifying the purpose of each item on the sheet
Job aid	Recommended Treatment Regimens for Uncomplicated Malaria in Brazil	Key information and general guidelines for dispensing antimalarials in Brazil after the confirmatory malaria diagnosis and based on G6PD enzyme activity
Comic book	Talking about Malaria: Exploring New Solutions to Treat Vivax Malaria	Comic book that explains the TRuST study, G6PD deficiency and new tools to fight the disease, such as the quantitative G6PD test and tafenoquine
Folder	Treatment of Patients with Vivax Malaria Symptoms	Information on the flow of care after routine implementation of the quantitative G6PD test and treatment for malaria, emphasizing the signs of hemolysis
Poster	Guidelines for Taking the G6PD test	Complete guidance on the quantitative G6PD test procedure including sample collection, preparation, reading, interpretation of results, and information on errors that may occur using the G6PD analyzer
Job aid	Health Worker Talking Points on G6PD	Key ideas for HCPs in guiding vivax malaria patients on G6PD testing and the new treatment

*Copies of some of the printed materials are available at https://www.vivaxmalaria.org/o-estudo-trust.

Following the two training phases, HCPs responsible for implementing quality assurance and supervision received additional instruction on how to perform quality control of the devices as recommended by the manufacturer. Also, they were shown how to review cases in which inconsistencies were detected and given guidance on how to investigate and report the reasons for discrepancies as well as the follow-up actions required, such as conducting specific training when necessary. For example, instances where patients were tested for G6PD twice due to a lack of HCP confidence or pipetting errors were documented to identify patterns which could indicate knowledge gaps that needed to be addressed. Thus, there was no fixed retraining schedule.

Because the HCPs came from diverse backgrounds with different knowledge levels and experience, their understanding and assimilation of the new tools and interventions varied considerably. Recognizing this, adaptations were introduced iteratively during the second phase and via reinforcing educational strategies following both training phases. These adaptations were designed based on the ‘RE-AIM’ framework, which emphasizes intervention evaluation in terms of reach, effectiveness, adoption, implementation, and maintenance [36–38]. Using this framework, interventions and educational strategies were tailored to address the specific needs and challenges HCPs encountered, with the aim of promoting outcomes that were sustainable in their setting.

### Qualitative data collection procedure

Face-to-face IDIs were conducted in comfortable and noise-free rooms within the health facilities, ensuring that the participants were at ease and not disturbed. FGDs and IDIs were led by FM, AP, JA, ES, AO, HG, FR, AS, and ER. Interviewers were previously trained and had experience with qualitative data collection. The average IDI duration was 35 minutes. FGDs comprised seven HCPs, one experienced mediator, and one observer. These were arranged at locations suitable for both the participants and the research team and were scheduled for about 90 minutes. For both IDIs and FGDs, a semi-structured interview guide was developed with nine open questions, supplementary questions, and instructions that allowed the interviewer to investigate the topic in more detail (Table 2). All qualitative data collection was done in Portuguese. To ensure complete anonymity, audio recordings were done for all IDIs and FGDs without personal identifiers for subsequent transcription. To enhance data reliability, both the mediator and observers recorded field notes during the FGDs, allowing for data triangulation.

**Table 2:** In-depth individual interviews and focus group discussion guide.

Questions	Objective
Could you describe the training you’ve received in detail? What did you like about the training? And what didn’t you like?	Evaluate the training offered to HCPs
If I gave you a G6PD test now, how would you feel about using it? What concerns would you have?	Assess the knowledge appropriation by these professionals
What do you think about refresher training?	Verify HCP confidence in the knowledge acquired in the training
How would you describe and evaluate the teaching material used in training?	Evaluate the materials training offered to HCPs
How did you find the classes? How do you feel about the organization of time during training?	Collect suggestions for improving training and knowledge for application in HCP work routines
How was the subject taught by teachers? Were you able to follow?	Evaluate the didactics of the teacher responsible for training
On leaving the training session what questions did you have regarding the G6PD test or regarding the new treatment for vivax malaria?	Evaluate the training offered to HCPs

### Data analysis

Data collection, analysis, and interpretation were performed simultaneously. After each IDI or FGD, audio recordings and field notes were reviewed. We conducted two rounds of categorization, the first in Excel and the second in MAXQDA software. The first round in Excel served as a pragmatic and accessible means to organize initial observations and impressions quickly. This preliminary categorization helped the researchers identify the major themes and patterns before delving into a more nuanced and detailed analysis using MAXQDA. The transition to MAXQDA allowed for a more comprehensive examination of the data, ensuring a thorough exploration of themes and categories through inductive and deductive coding. Two researchers (FM and AP) collaborated to build a consensus on the categories. Additionally, three researchers (MF, AP, and FR) developed a code book. During the thematic analysis, comparisons were established among interviewed HCPs and facility type to identify differences and similarities in perceptions. Data analysis continued until inductive thematic saturation was achieved.

### Research team and reflexivity

The qualitative research team comprised seven PhD qualitative researchers, two females (AS, DB) and three males (FM, WM, MB), with expertise in neglected tropical diseases, and two males (ML and DP) with experience in clinical research and malaria case management. FM, AS and WM had previously conducted qualitative research with malaria healthcare providers in the Amazon region [23,39]. The study team had no prior relationship with the participants.

## Results

### Participant characteristics

Overall, 115 HCPs participated in the study, with 94 completing IDIs and 21 participating in three FGDs (Table 3).

**Table 3:** Characteristics of HCPs participating in the study.

Characteristic	Phase one: higher-level facilities			Phase two: lower-level facilities		
	Porto Velho	Manaus	Total	Porto Velho	Manaus	Total
Number of HCPs	26	28	54	27	34	61
Gender						
Female	21	15	36	17	8	25
Male	5	13	18	10	26	36
Education level						
High school	8	7	15	16	30	46
College	18	21	39	11	4	15
Work location						
Urban	26	18	44	15	3	18
Peri-urban	0	10	10	12	31	43
Job function						
Field agent	0	2	2	5	25	30
Physician	10	9	19	5	0	5
Microscopist	5	3	8	10	6	16
Nurse	4	1	5	4	0	4
Pharmacist	4	4	8	1	0	1
Laboratory technician	2	2	4	0	0	0
Biochemist	0	7	7	0	0	0
Supervisor	0	0	0	1	2	3
Notifier	0	0	0	1	1	2
Nursing technician	1	0	1	0	0	0

### Theme 1: Experiences of the two training phases

#### Subtheme 1.1: HCPs vigilant about the risk of acute hemolytic anemia

Both training sessions successfully increased awareness of AHA signs following 8-aminoquinoline use. Several HCPs reported unfamiliarity with AHA signs and symptoms before training. However, following training, all HCPs were able to list dark urine as an important clinical sign indicating AHA, and most could identify yellow skin and eyes. In relation to AHA case management, interviewees were aware of the appropriate action, especially referring patients to higher-level facilities.

*“The yellowish skin*, *the dark urine*, *but these effects are only after the patient takes the medication and if he presents the symptoms*, *we also put it on the SIVEP form*.*"* (Participant 30, field agent, Manaus, phase two).

*“If the wrong treatment is given to the patient*, *this patient may experience symptoms that can harm the patient such as*: *coffee-colored urine*, *vomit*, *become yellowish*.*"* (Participant 27, nurse, Porto Velho, phase two).

In phase one, some physicians associated the G6PD test with tafenoquine only and were unaware of the benefits in preventing AHA following primaquine. Similarly, other HCPs were more inclined to prioritize G6PD testing prior to tafenoquine than before primaquine administration, with a limited understanding that the G6PD test affected primaquine prescription. Consequently, in phase two, greater emphasis was placed on educating participants about the AHA risk associated with both 8-aminoquinolines in G6PD-deficient patients and the impact on treatment.

*“[*…*] If it [the G6PD level] goes below 6*, *it’s not 7*, *it’s 6*, *because I don’t have my notes here*, *he [the patient] can’t take tafenoquine*. *He’s not able to take tafenoquine because he doesn’t have this protective enzyme*. *He can have hemolysis*, *right*? *[*…*]*.*”* (Participant 21, biochemist, Manaus, phase one).

*"Prior to implementation*, *we did not have information about the patient’s hemoglobin level and G6PD status*, *resulting in the drug dosage being determined only by the patient’s weight*. *This approach carried the risk of administering excessive doses of primaquine*, *as*, *for example*, *there were cases where a G6PD deficient patient could be given up to 21 pills for 7 days*. *In such cases*, *hemolysis of the red blood cells would occur*, *leading to potentially serious consequences for the patient*. *Thus*, *the introduction of G6PD testing has proven to be instrumental in improving processes for relapse and treatment monitoring*.*"* (Participant 06, pharmacist, Manaus, phase one).

#### Subtheme 1.2: Limited success in single-round training

In the case of physicians, some participated in online training and others claimed that they only received supporting materials. Reasons for missing the online training included work demands, shift schedules, and staff turnover. However, doctors who were trained online generally classified the training videos as good, explanatory and didactic, though more technical information was needed. No modifications were made to physician training in phase two as this phase was directed at non-physicians. However, face-to-face follow-up training was conducted where physicians had found the online training inadequate.

*“This online course business—not everyone does it*. *It’s different from the person coming here to talk to us*, *you know*?*"* (Participant 31, doctor, Porto Velho, phase one).

*"Look*, *in-person classes everyone pays attention*, *unlike online classes*.*"* (Participant 42, doctor, Porto Velho, phase one).

*“The first training was by videoconference*, *then a doctor came from CEMETRON*, *and attended here*. *She explained and gave a class about what it was and how it was done*. *Gave us an update*. *I found this second moment complementary because*, *at first*, *we understood*, *then during professional practice*, *some doctors did still not understand*.*”* (Participant 32, doctor, Porto Velho, phase one).

For the non-physician training sessions, across both phases there was a consensus among HCPs that more than one training day was needed to consolidate the new knowledge, especially regarding the G6PD testing procedures. The HCPs most involved with malaria diagnosis, such as biochemists and microscopists, considered the practical classes essential for learning and were confident in performing the quantitative G6PD test after training. However, they requested more hands-on training opportunities to embed confidence in performing the test. Some HCPs who use diagnostics in the field reported that they felt the need for additional practical training and reported that learning how to perform the G6PD test occurred after the training, during the work routine. They reported that there were some colleagues who still felt insecure in doing the test because of limited opportunities to practice. It was suggested that training duration should be longer with retraining to reinforce learning.

*"I felt that the training was brief because we only had one opportunity to interact with the tools*. *So*, *my understanding of the material is quite basic*.*"* (Participant 05, biochemist, Manaus, phase one).

*"In my point of view*, *the training was very fast*. *It should be more in-depth*, *longer*, *with more information-rich content for us to have a little more confidence*. *We should have a new training*, *a specific training for the test*.*"* (Participant 22, microscopist, Manaus, phase one).

#### Theme 2: Primary insecurities reported by HCPs after training

After training, the main operational errors occurred during the G6PD test execution, leading to insecurities in competently conducting the test. Unlike their counterparts in phase one who are based in hospitals, the HCPs in phase two work in the field and are engaged in external activities. They visit people infected with malaria, actively participate in outreach efforts to identify and address cases in diverse settings, and have fewer colleagues and resources that they can immediately draw on. Those working in the field reported greater insecurity than those working in clinics, as did those with a longer interval between training and implementation.

HCPs’ doubts regarding the G6PD test included turning the equipment on, the time limit to transfer the sample to the test strip, the need to redo the test, and the interpretation of results (Table 4). The most frequently reported error was improper pipetting. For the sample preparation, a pipette is used to collect a drop of capillary blood from a finger prick and mix it with the test reagents. During this stage, errors occurred due to insufficient blood sample collection or homogenization, leading to excessive air bubbles, resulting in an incomplete mixture. HCPs also felt increased insecurity because the manufacturer provided a specific number of pipettes; i.e., two for each test, one for blood sampling and one for sample transfer from the buffer tube to the test strip. From their perspective, this did not allow for any errors or retesting. Most HCPs reported the need for a greater number of pipettes in the kit. Despite this limitation, where mistakes were noticed or the result was G6PD-deficient, all HCPs reported repeating the procedure to ensure patient safety. Repetition and co-worker help and conversations alleviated these insecurities.

**Table 4:** The primary uncertainties in handling the G6PD test.

Primary insecurities	Quotes
Turning on the test	*“The first time I trembled*. *I trembled with fear of making a mistake*, *but I did not make a mistake*. *I forgot how to turn on the device [*.* *.* *.*]*" (Participant 39, microscopist, Porto Velho, phase two).
The time limit to transfer the sample to the test strip	*“The test had to be redone once*, *because it took me a long time to get the reagent and put it in the apparatus*. *So*, *they told me if you delay*.* *.* *.*you might get a different result*. *So*, *I only used the device once*.* *.* *.*"* (Participant 46, microscopist, Porto Velho, phase two). *"The test needs to be redone if*, *at any point*, *you encountered any setbacks and ended up leaving the sample in the pipette for a period that exceeds the time required for insertion into the test apparatus*. *In this case*, *it is necessary to discard that sample and conduct a new test*. *Sometimes*, *the device may experience a reading error*, *and automatically*, *you must redo the test*, *collect a new sample*, *and renew the process*.*”* (Participant 25, microscopist, Porto Velho, phase one).
Redo the test and insecurity about the result	*"The girls here*, *when they were starting to do the test*, *I always asked them to do it again*. *I asked one of the girls to repeat the activity and not to give more than 6*, *right*? *To see if it gave a kind of weird result*, *I always asked them to repeat it*, *so we could have some notion like*: *did it take too long*? *or maybe it wasn’t a mistake at the time of doing it*? *you know*? *It happens sometimes*, *it has happened that we do it*, *I asked a colleague to do it again for me*, *because I think I took too long to do it*, *you know*? *Because that part of the test is the most annoying like that*.*"* (Participant 43, microscopist, Porto Velho, phase one). *“It has already given out two errors in the performing of the test*. *One involved a male patient*, *and the result was 0*.*0 U/gHb*, *so I said*, *‘Calm down*. *Calm down*. *Let’s do another test*.*’ It cannot [give this result]*, *then it was normal*, *it was 6*.*4 U/gHb*.*”* (Participant 29, field agent, Manaus, phase two).
Mistakes in front of patients	*“In the beginning*, *I had a lot of difficulties [*.* *.* *.*]*. *Even in doing the step by step [of the G6PD test]*. *For example*: *’it went wrong because of the time it spent on the device’*. *So*, *until I learned how to do the exam properly*, *it was not a very good situation*, *I was nervous because a patient was waiting [to be seen]*, *we had to do the exam*. *But anyway*, *I think it’s a matter of time and adaptation*.*”* (Participant 08, microscopist, Porto Velho, phase one). *"The only problem with the test is that sometimes it has errors*. *There have been some mistakes*, *and we had to retest*. *This creates insecurity in people*. *There is that doubt*: *’Is the result of the second test really the value of G6PD that the patient presents*?*"* (Participant 35, pharmacist, Manaus, phase two).
Pipetting processes	*"It’s possible to do it even with the kit for us*, *it comes with that test strip*, *right*? *It’s the test strip*, *the device*? *those*.* *.* *. *the solutions*? *And the pipettes*, *including the pipettes*, *I don’t know how to use them because they don’t need to suck*.* *.* *.*"* (Participant 06, pharmacist, Manaus, phase one). *“And there are some things they told us*, *it’s all counted*, *you can’t miss a pipette of it*. *If you miss one*, *you must write it down*. *I’m even scared*! *And if suddenly [a pipette] falls to the ground*, *you must use another one*, *I’m afraid*. *It’s something like that*, *a lot of responsibility with that device*, *with the tapes*. *If you use more than 2 or 3*, *you must write down the reason*.” (Participant 51, microscopist, Porto Velho, phase two).

HCPs attributed their errors in conducting the G6PD to the few practical classes they received, lack of practice because of a decline in malaria cases shortly after implementation, a change in routine, and unfamiliar procedures. Also, HCPs noted the differences between the laboratory-based training and the field environment, such as the physical environment and the potential for embarrassment when making mistakes in front of patients. The field environment is dynamic and frequently unpredictable, introducing distinctive challenges that cannot be easily reproduced in the controlled setting of a laboratory. Factors such as weather conditions, varying terrains, and the necessity to conduct tests on irregular surfaces, such as the seat of a motorcycle, have significantly contributed to this perception. However, they also noted that their performance improved with practical real-life repetition over 15 days as the new procedures were integrated into their work routine.

*“Initially*, *during the first week of using the new tools*, *mistakes were common due to the unfamiliarity*. *However*, *over time*, *we started to understand better*, *and team members helped each other out by resolving doubts and answering questions*. *We organized morning meetings to clarify any remaining doubts*. *Now*, *collecting samples has become quick and efficient*, *and no one gets confused*. *We even organized ourselves to learn the names of the reagents and components*, *so we can confidently respond when someone asks*.*”* (Participant 46, microscopist, Manaus, phase two).

In Porto Velho, some HCPs reported insecurity and requested retraining because implementation was interrupted at higher-level facilities. In Manaus, where training and implementation were synchronous, the need for immediate retraining was perceived as less important than in Porto Velho, but still necessary to maintain skills and develop knowledge.

*“[*…*] Within our malaria care program*, *I think we would always need to have a retraining*, *let’s retrain*, *retraining in the sense that we are always going over*.*”* (Participant 10, biochemist, Porto Velho, phase one).

### Theme 3: Fragmented knowledge identified after training

After phase one training, some HCPs believed that tafenoquine and the quantitative G6PD test were still being assessed for efficacy and safety, commonly using expressions like ‘*new drug*’ and ‘*drug in testing*’. Such scientific misperceptions could negatively affect HCP and patient acceptance of tafenoquine and quantitative G6PD testing. To mitigate this issue, expressions such as ‘new drug’ were avoided in phase two, whereas Ministry of Health approval for tafenoquine and the quantitative G6PD test was emphasized. After phase two training, including the incorporation of recommended adjustments, it was found that only two HCPs still did not fully comprehend that tafenoquine had completed testing and was approved for clinical use in Brazil.

*"I do believe that we are indeed conducting research with tafenoquine to see if it really works*. *I am not sure at which stage this test is*, *but I believe it is still being tested*.*"* (Participant 09, nurse, Manaus, phase one).

One concerning observation made after phase one was that just one of the interviewees mentioned breastfeeding as a consideration for 8-aminoquinoline treatment. This was despite all HCPs recognizing the importance of a differentiated treatment for pregnant women. In phase two, appropriate treatment in pregnant and lactating women was emphasized. Consequently, all HCPs interviewed after phase two understood that the treatment of pregnant/breastfeeding women was different even when a G6PD result was acceptable for tafenoquine or primaquine treatment.

*"In the case of a lactating woman who is breastfeeding up to one month*, *she cannot be treated with tafenoquine*. *We will look for another treatment for her*, *okay*?*"* (Participant 36, microscopist, Porto Velho, phase one).

### Theme 4: Strategies employed to improve learning outcomes

#### Sub-theme 4.1: *In situ* reinforcement training

According to HCPs, some lower-level healthcare units received *in situ* reinforcement training aimed at addressing operational errors. Local health authorities made this decision and conducted specific training tailored to the setting and specific issues that HCPs were facing. Some HCPs in both municipalities had more than one meeting with the training team to clarify doubts regarding the G6PD test, including one-on-one counselling, which increased their confidence when performing the test and prescribing treatment. They stated that this *in situ* monitoring and retraining process contributed to a better understanding of the implementation process.

*“When I went to the training session*, *I already went with my doubts already written down*, *you know*? *So*, *I was clarifying my doubts there at the time*. *So*, *it was very easy because*, *at the same time*, *she answered my questions*.*”* (Participant 28, nurse, Porto Velho, phase one).

#### Sub-theme 4.2: Informative and educational materials

Providing informative/educational material for prior reading was also cited as a measure to improve learning during training (Table 1). In phase two, HCPs could review the content in advance, familiarize themselves with the concepts, terminology, and procedures covered, and prepare queries in advance.

*“They were supposed to deliver the didactic material before giving the training so that we could have a look and see how the test is handled and we would learn more*.*”* (Participant 36, microscopist, Porto Velho, phase one).

*“The training was previously scheduled*, *and they sent the manuals in advance for us to read*, *this helped us to write down any doubts in advance so that the training would last*, *in addition to the theoretical and practical classes*, *we asked them for examples of real situations with the G6PD test and tafenoquine [*…*]*.*"* (Participant 52, microscopist, Porto Velho, phase two).

#### Sub-theme 4.3: Group conversation approaches

In Porto Velho, two healthcare units that were experiencing problems with adherence to the new procedures applied talking circle and talking wheel approaches. Both techniques assume that everyone involved has valuable insights to share. In the talking circle, communal learning and open dialogue is encouraged by each participant taking a turn to speak while others listen attentively without interruption. The aim is to foster shared goals and respect, with the facilitator encouraging reflection and discussion between participants [40]. In the talking wheel approach, the instructor acts as the hub, steering the discourse while encouraging active participation and critical thinking. Students become spokes on the wheel, contributing to discussions, posing questions, and engaging in collaborative exercises. In this case, a practical class on conducting the G6PD test was incorporated into the session, encouraging cooperation and group-led problem solving. These group conversation approaches were well received by HCPs, who reported that the discussions helped them identify errors in G6PD test execution. The exercises also addressed their insecurities about the test and answered their questions regarding issues that remained unclear after their initial training.

*"The talking circles were truly eye-opening for us*. *It felt like a safe space to share our experiences and learn from each other*. *I now have a much clearer understanding of the G6PD test*.*”* (Participant 82, supervisor, Porto Velho, phase two).

#### Sub-theme 4.4: Peer-to-peer information exchange

After phase one training, HCPs created a WhatsApp group to exchange information, doubts, and solutions. HCPs proposed this initiative with local government support. According to the HCPs, it was the best strategy for promoting adherence and integration for the novel approaches in *P*. *vivax* malaria case management. Furthermore, the group allowed real-time dissemination of educational materials developed after the training and important updates. These health educational materials were created based on feedback from the training sessions and respondents considered them an important strategy to strengthen knowledge. For example, short videos (S4 File) and cards (S5 File) were created and distributed via WhatsApp.

*"We created a group to chat in that period of improvement of studies*, *when they passed on the knowledge to us*. *Regarding that*, *it was great*. *I liked it very*, *very much*. *Their willingness to answer questions on any issue*.*”* (Participant 11, doctor, Porto Velho, phase one).

“*I really liked the videos that were shown on WhatsApp*, *it’s easier to understand what we end up forgetting*.” (Participant 63, field agent, Manaus, phase two).

### Training approach

**Fig 3:**
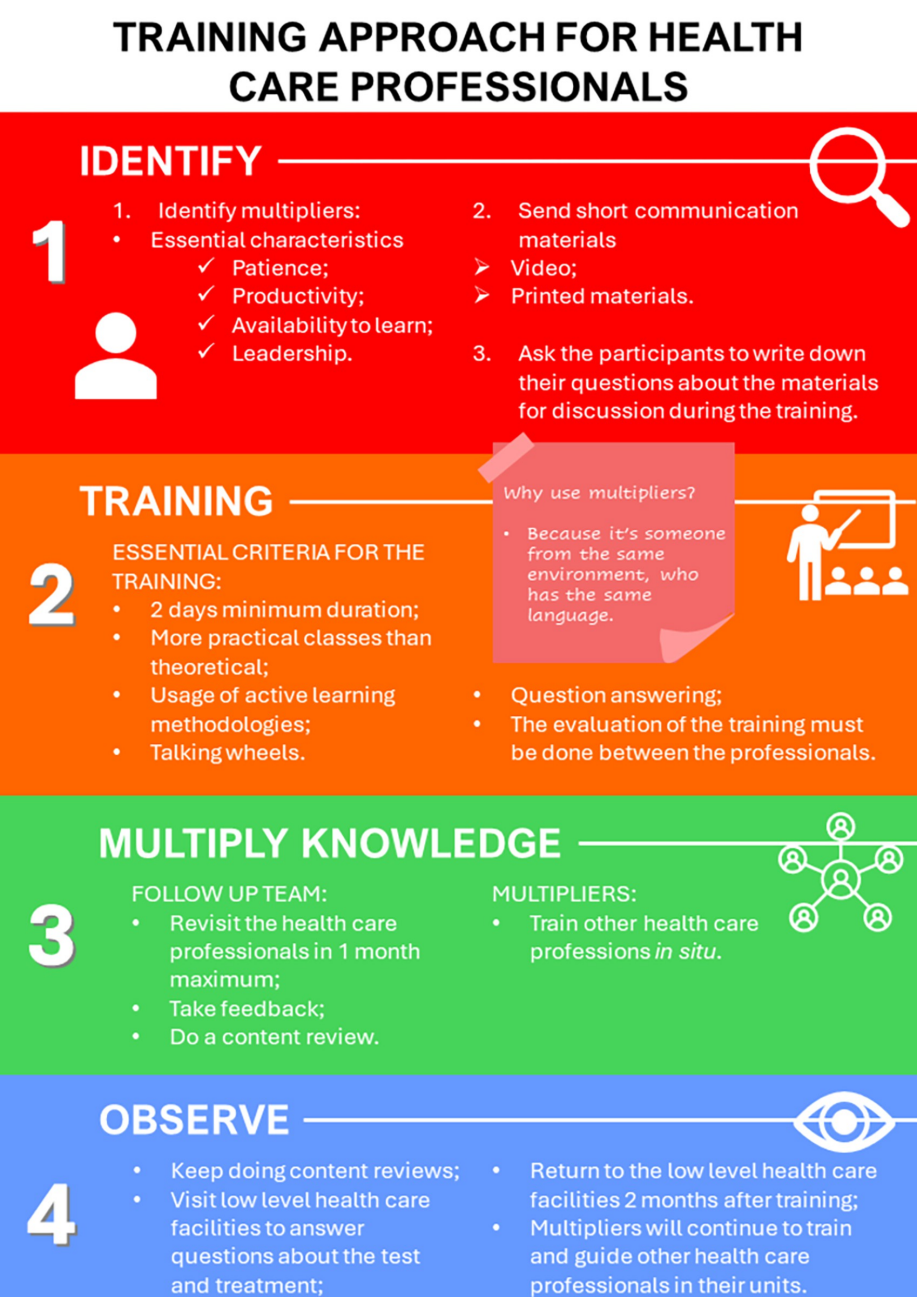
Proposed training approach for future training in the implementation of tafenoquine and the quantitative G6PD test, based on the feedback from HCPs. F. Murta, created in Canva.

HCP experiences with the training sessions and the resulting iterative adaptations directed the development of a training approach. This aimed to support future implementation of quantitative G6PD testing and tafenoquine across the Brazilian healthcare system (Fig 3). The training approach was designed to address the challenges HCPs identified during their practice and provide them with the necessary tools and resources to overcome them. It incorporates best practices and guidelines for quantitative G6PD testing and tafenoquine usage, as well as practical tips and strategies for dealing with everyday issues encountered during clinical practice. The training approach was designed to be flexible and adaptable to promote effective implementation in a variety of healthcare settings and across the diverse range of HCP knowledge and experience.

The key concept proposed in the training approach is the identification of expert users with competence in quantitative G6PD testing and effective assimilation of the treatment algorithm. This proposal was formulated based on the favorable feedback from some HCPs in Porto Velho following conversation-based retraining efforts (talking circles/wheels). Additionally, it involves identifying key HCPs within each group that are frequently relied upon by their peers for resolving inquiries and uncertainties. These individuals serve as multipliers by actively disseminating their knowledge and expertise among their peers, thus fostering a collaborative and knowledge-sharing strategy. Primarily nominated from within their group, and with good understanding and practice in administering the G6PD test and prescribing 8-aminoquinolines, multipliers already hold the respect and trust of their colleagues. Most importantly, multipliers must have a helpful and supportive attitude.

*"[*…*] I had a lot of doubts about the G6PD test*, *and I used to ask my fellow HCP who learned more quickly a lot of questions*. *So*, *when I’m unfamiliar with a procedure*, *I always turn to him*, *especially regarding the new malaria treatment*, *which*, *in my opinion*, *is the most complex part of the new routine*.*"* (Participant 46, microscopist, Manaus, phase two).

The development of the training approach represents a significant advancement for implementation of *P*. *vivax* radical cure within the Brazilian healthcare system. It was designed drawing upon the experiences and insights of HCPs, whose direct feedback played a pivotal role at every stage of its development. Thus, the training approach is well-equipped to address the barriers identified by our participants, thereby enhancing the success of the implementation process. The training approach is now poised for implementation and validation by the Ministry of Health in the forthcoming years, extending its applicability to other municipalities that implement quantitative G6PD testing and tafenoquine.

## Discussion

This study evaluated HCP perceptions and experiences following training to support implementation of a new treatment algorithm for *P*. *vivax* radical cure incorporating quantitative G6PD testing and single-dose tafenoquine in the Brazilian Amazon. This required HCPs to master the technical requirements of conducting the point-of-care quantitative G6PD test and develop an understanding of the relevance of the G6PD test results to select appropriate treatment with tafenoquine, daily primaquine, weekly primaquine, or chloroquine alone. HCPs had to change their practice to systematically perform the G6PD test and apply the test result, take measures for pharmacovigilance, including patient counselling, and complete the updated SIVEP-Malária forms. These requirements demanded the acquisition of technical and medical knowledge as well as the application of interpersonal skills. Moreover, HCPs had to integrate their acquired knowledge and skills into their daily work routines.

Many of the difficulties HCPs experienced concerned the practical aspects of conducting the quantitative G6PD test, and there needs to be sufficient focus on skills acquisition and building confidence, including *in situ* training tailored to the setting. Similar to the findings of this study, in Bangladesh, although laboratory technicians appreciated the portability and ease of use of the G6PD test, some found the sample preparation procedure complex, anticipating issues with insufficient blood samples, difficulties with the pipette, and bubble aspiration during periods of intense workflow [41]. In Cambodia, HCPs tasked with conducting the G6PD test as part of their daily responsibilities also expressed apprehensions related to the pipetting process [42]. These concerns encompassed the use of two pipettes, and difficulties in mixing the blood sample with the buffer tube. Nevertheless, as observed here, there was a gradual enhancement in proficiency with the equipment, attributed to regular monthly training sessions and ongoing supervision [42].

In our study, the training program used an interactive approach with theoretical and practical sessions, consistent with World Health Organization recommendations for training microscopists in malaria diagnosis [43]. In the first training phase, theoretical and practical instruction was conducted in two separate sessions. However, feedback led to closer integration of theoretical and practical activities into short 15-minute sequential sessions in phase two. This modification was well accepted by the HCPs as theoretical information was immediately applied and thereby reinforced. However, there was also consistent feedback from HCPs that the fundamental changes to *P*. *vivax* malaria case management and the required practical skills could not be addressed sufficiently in a single training day. The initial decision to condense training into one day was based on the practicalities of delivering training to 659 HCPs within a short timeframe without disrupting malaria services. Also, a one-day format is frequently adopted in Brazil for HCP training, though it is acknowledged that this is not always appropriate [44]. However, in a previous mixed-methods study of G6PD test implementation in Brazil, a single 4-hour theory and practical training session was considered adequate [23]. Also, a study conducted in Brazil, Ethiopia, and India showed that the skills to conduct the point-of-care G6PD test and interpret the results could be acquired in as little as three hours [45]. However, in these previous studies, only primaquine administration was considered, whereas in the current study the treatment algorithm incorporated tafenoquine and primaquine. This increases the complexity of interpretation of the G6PD results and represents a completely different treatment paradigm for *P*. *vivax* malaria. Thus, to increase HCP confidence in both conducting the G6PD test and applying the treatment algorithm, we strongly recommend that future training sessions for implementation are conducted over a minimum of two days. The first day would be a standard theoretical and practical format, as evaluated in this study, and the second day would follow-up with training closer to the HCP work setting, in line with their field conditions and oriented to their specific needs and concerns.

There were no prespecified plans to conduct refresher training, but HCPs suggested that this would be useful and where additional training was conducted, it was perceived to be valuable. This was also identified in a previous study of quantitative G6PD testing prior to primaquine, with regular refresher training particularly needed in locations with lower malaria caseloads [23]. Similarly, in Bangladesh, refresher training was perceived necessary because the low prevalence of *P*. *vivax* malaria would lead to the loss of skills over time [41]. During implementation of the G6PD test in Vietnam, although proficiency in administering the test was high after the initial training, skills did erode, primarily because of the low case volumes, and a 3-month cycle of refresher training was recommended as well as ongoing supervision [46]. Thus, it is crucial to consider periodic on-the-job training and consistent supervision to enable HCPs to enhance their skills progressively and to maintain them as case numbers decline.

Within the study, several strategies were developed following the IDIs and FGDs to support continued learning and skills development and to bridge gaps in knowledge following the initial training. Formal retraining was conducted specifically in the units where HCPs lacked confidence, and this was a successful strategy for consolidating learning. Associated with this, another effective measure was the counseling visits and the conversation circles or talking wheel approaches. The conversation circles (also known as yarning circles) were an important tool for fostering horizontal dialogues with participants, thus creating a safe space to share experiences, emotions, and sensitive information [47]. Rooted in indigenous practices, the aim is to identify the social helping relationships between participants within the wider context of their work in the community [48]. Communication is non-linear, emphasizing equal participation and the establishment of a trustful relationship. This is a significant departure from the top-down hierarchical systems of learning but was particularly valuable in integrating experiences in the field, examining and overcoming anxieties and solving problems collectively. This approach recognized that despite the different job roles and educational levels taking part in the training, all participants had challenges, and all could learn from one another to ensure better outcomes for patients. To our knowledge, this is the first use of such an approach for HCP training to institute a new treatment paradigm, though it has been used for patient consultation, particularly in indigenous populations [49–51].

An additional innovation within the study was the use of peer-to-peer learning using WhatsApp groups. This was convenient and supportive for HCPs and allowed rapid dissemination of specific information for learning reinforcement and response to queries. WhatsApp is the most widely used communication app among smartphone users worldwide, particularly in Brazil, which has approximately 147 million users. Despite its ubiquity, there are few published reports of the intentional use of this platform to deliver malaria services. Examples include cross-border coordination of *P*. *vivax* outbreak responses between Indian and Bhutan health authorities [52], combatting misinformation in Sierra Leone [53], sending patient follow-up reminders in Brazil [54], supporting patient advice on travel hazards [55], and providing remote medical consultations for military personnel [56]. However, there is only one report where WhatsApp has been used to facilitate implementation; in Mozambique, it became a vital channel during a universal mosquito net coverage campaign, providing support to district and provincial teams and fostering information sharing among members [57]. The authors suggested that it could also be used to monitor activities and post-implementation mobilization continuously [57]. Despite the scarcity of studies on its application in healthcare, especially in training, implementation, monitoring, and supervision, our findings, combined with those from Mozambique, indicate WhatsApp’s potential as a direct communication channel for frequently asked questions, information sharing, and experiences, highlighting its value as a support tool for implementation processes.

In this study, the trainings were especially effective in increasing awareness of AHA signs and symptoms, which was new information for many HCPs. In fact, HCPs were so concerned about safety that at the beginning of the implementation they repeated the G6PD test whenever the value was below 6.1 Ug/Hb (G6PD intermediate/deficient). However, after a few months most HCPs were confident with low G6PD enzyme activity results. The tendency for repeated testing during initial implementation should be considered in cost-effectiveness studies and incorporated into quantification and budgeting forecasts.

The difficulty in comprehending the significance of G6PD enzyme activity and its importance in malaria treatment initially presented a barrier for HCP understanding. Importantly, G6PD status was not previously considered within malaria case management and so was completely unfamiliar to HCPs. G6PD deficiency is uncommon in Brazil with a prevalence of around 6% [22], making it difficult for HCPs to understand its relevance for malaria treatment. However, following training, all HCPs had high awareness of the need for quantitative G6PD testing prior to tafenoquine. This was demonstrated in the TRuST feasibility study, with 100% (2685/2685) adherence to quantitative G6PD testing prior to tafenoquine [32]. Thus, the challenges to understanding could be overcome with daily practice, consistent reinforcement, and supportive learning, such as short videos and WhatsApp cards. However, communicating the need for quantitative G6PD testing prior to primaquine was more challenging. HCPs had previously prescribed primaquine without any G6PD testing (qualitative or quantitative) [58], and changing old habits was more difficult than instilling new ones. This was also noted in a previous study evaluating a qualitative G6PD test in Brazil prior to primaquine, with most HCPs unaware of G6PD deficiency and primaquine-related adverse effects [39]. Similarly, evaluation of the same quantitative G6PD test used in the current study prior to primaquine showed that HCPs and patients did not always associate AHA signs and symptoms with malaria treatment [23]. Nevertheless, primaquine can be associated with significant adverse effects [59], and quantitative G6PD testing is highly cost-effective in avoiding primaquine-associated hospitalizations for AHA [24]. In the current study, although the need for quantitative G6PD testing prior to primaquine was emphasized during phase two training, the feasibility study showed 91.5% (2954/3228) adherence to quantitative G6PD testing prior to primaquine [32]. Consequently, continued evaluation, monitoring, and support is required to identify and address this knowledge gap.

In addition to G6PD status, age, pregnancy, and breastfeeding status are all features of the new treatment algorithm. All HCPs in phase one and two were aware that pregnant women were excluded from 8-aminoquinoline treatment. Consistent with these findings, the feasibility study showed that no pregnant women were prescribed 8-aminoquinolines [32], probably because this did not represent a change to current practice. Patient age was a new consideration within the treatment algorithm but was absorbed into practice consistently [32]. However, behavior change is required around determining whether women *P*. *vivax* patients are breastfeeding and the duration of breastfeeding. This was poorly appreciated in phase one, but in phase two all HCPs were able to identify breastfeeding as a consideration for 8-aminoquinoline treatment. However, the feasibility study indicated that obtaining and/or recording this information was insufficient [32]. Thus, despite adaptations to phase two training, the need to determine breastfeeding status was not fully addressed, and there is scope to further examine the reasons for this gap in knowledge and practice and design specific additional interventions to support HCP and patient communication around breastfeeding status.

Tafenoquine is perceived as a valuable new tool for addressing *P*. *vivax* malaria [60], and implementation studies are being considered or conducted in several countries [41,61,62]. QualiTRuST was conducted to support quantitative G6PD testing and tafenoquine implementation within the Brazilian health system. The Amazon River basin encompasses several countries which face similar challenges in addressing *P*. *vivax* malaria [58], and study findings are generalizable across the region. However, the results may not be generalizable to other malaria endemic countries as *P*. *vivax* transmission dynamics, health system organization, historical practices, and social and cultural differences will determine the training required. For example, in Bangladesh, a key barrier to quantitative G6PD testing adoption is the diminishing importance of malaria as perceived by both policy makers and the affected communities [41]. In contrast to Brazil, where HCPs overestimated primaquine safety, in Asia there are historical concerns among HCPs regarding primaquine safety without G6PD testing [63]. Although these concerns could be addressed by systematic quantitative G6PD testing, introducing this intervention may require a different approach to that adopted in Brazil. However, regardless of the specific training model adopted, it is necessary to establish an easily accessible communication channel between frontline HCPs for continued peer-to-peer support and to provide real-time feedback on performance issues and knowledge gaps that were not addressed during training.

Study strengths include the diversity of participants’ backgrounds and job roles and the iterative, rapid development process which allowed ongoing modification and evaluation of the training processes. However, given the large scale of the implementation across a wide variety of settings over a short period of time, pragmatic decisions were made to simplify the study design and two limitations are noted: (1) the use of random sampling, whereas purposive sampling with gradual selection based on participant characteristics may have ensured maximum variation, enhanced data saturation, and included more varied perspectives, and (2) the absence of participant observation, which is recommended for a more comprehensive understanding during IDIs and FGDs.

## Conclusion

For Brazil to achieve malaria elimination, successful implementation of quantitative G6PD testing and single-dose tafenoquine for *P*. *vivax* radical cure is critical. In Brazil, tafenoquine clinical efficacy and safety was evaluated as part of a multi-national clinical trial program [16,17], with subsequent assessment of the operational feasibility and acceptability of the quantitative G6PD test [23]. The encouraging findings prompted evaluation of the operational feasibility and acceptability of the revised treatment algorithm incorporating quantitative G6PD testing and tafenoquine deployed together [32].

This study analyzed qualitative data from HCPs who received training on quantitative G6PD testing and tafenoquine implementation. The findings were used to direct adaptations to the local context and setting, and drive improvements, including the development of supportive communication strategies and continuing education initiatives to accelerate and solidify HCP learning. This process supported the development of a training approach for roll out of these new tools across the Brazilian health system. This training approach can also be used as a starting point for subsequent modifications to the *P*. *vivax* treatment algorithm, for example, the potential extension of tafenoquine for use in children [64]. By embracing new treatment options for *P*. *vivax* malaria and taking proactive measures to ensure their safe and effective use, Brazil provides a template for other Latin American countries to follow in the fight against this high-burden disease, especially in the Amazon.

## Supporting information

S1 File Consolidated Criteria for Reporting Qualitative Research Checklist. (DOCX)

S2 File Content proficiency assessments. (DOCX)

S3 File Lesson plan for the second training phase. (DOCX)

S4 File Shorts videos for mobile phone. (MP4)

S5 File WhatsApp cards. (DOCX)
