# Supplementary material for: Perspectives of healthcare professionals on training for quantitative G6PD testing during implementation of tafenoquine in Brazil (QualiTRuST Study)
Source: PLoS Negl Trop Dis. 2024 Jun 5;18(6):e0012197. doi: 10.1371/journal.pntd.0012197 (PMC11152287; doi:10.1371/journal.pntd.0012197)
Supplement: S1 File — (DOCX) [file pntd.0012197.s001.docx]

**Consolidated criteria for reporting qualitative studies (COREQ): 32-item checklist**

Developed from:

Tong A, Sainsbury P, Craig J. Consolidated criteria for reporting qualitative research (COREQ): a 32-item checklist for interviews and focus groups. *International Journal for Quality in Health Care*. 2007. Volume 19, Number 6: pp. 349 – 357

| **No. Item** | **Guide questions/description** | **Reported on Page #** |
| --- | --- | --- |
| **Domain 1: Research team and reﬂexivity** |  |  |
| *Personal Characteristics* |  |  |
| 1. Inter viewer/facilitator | *Which author/s conducted the interview or focus group?*  FGDs and IDIs were led by FM, AP, JA, ES, AO, HG, FR, AS, and ER. | Qualitative data collection procedure, 15. |
| 2. Credentials | *What were the researchers’ credentials?*  PhD qualitative researchers. | Research team and reflexivity, 17. |
| 3. Occupation | *What was their occupation at the time of the study?*  Physicians, university professors, and qualitative researchers. | Title page. |
| 4. Gender | *Was the researcher male or female?*  Two females and five males. | Research team and reflexivity, 17. |
| 5. Experience and training | *What experience or training did the researcher have?*  Renowned researchers with experience in qualitative research. | Research team and reflexivity, 17. |
| *Relationship with participants* |  |  |
| 6. Relationship established | *Was a relationship established prior to study commencement?*  The study team had no prior relationship with the participants. | Research team and reflexivity, 17. |
| 7. Participant knowledge of the interviewer | *What did the participants know about the researcher? (e.g., personal goals, reasons for doing the research).*  Participants were briefed on the purpose of the study and understood that it was a research project. Educational ethical approval had been granted and participants reviewed the participant information documentation prior to giving their written informed consent to be involved. | Ethics statement, 7. |
| 8. Interviewer characteristics | *What characteristics were reported about the inter viewer/facilitator? e.g., bias, assumptions, reasons and interests in the research topic.*  No interviewer-related biases were identified. | Qualitative data collection, 15. |
| **Domain 2: study design** |  |  |
| *Theoretical framework* |  |  |
| 9. Methodological orientation and Theory | *What methodological orientation was stated to underpin the study? e.g., grounded theory, discourse analysis, ethnography, phenomenology, content analysis.*  Thematic analysis and categorization were conducted through inductive and deductive coding. | Data analysis, 16 |
| *Participant selection* |  |  |
| 10. Sampling | *How were participants selected? e.g., purposive, convenience, consecutive, snowball.*  The purposeful sample size was based on the principle of saturation, where in-depth individual interviews (IDIs) and focus group discussions (FGDs). | Sampling, 9. |
| 11. Method of approach | *How were participants approached? e.g., face-to-face, telephone, mail, email.*  We did a face-to-face interaction with HCPs. | Qualitative data collection procedure,15,16. |
| 12. Sample size | *How many participants were in the study?*  115 HPs | Participant characteristics, 17, 18. |
| 13. Non-participation | *How many people refused to participate or dropped out? Reasons?*  No one has given up on participating in the project. | N/A |
| *Setting* |  |  |
| 14. Setting of data collection | *Where was the data collected? e.g., home, clinic, workplace.*  IDIs and FGs were arranged at workplace in locations suitable for both the participants and the research team and were scheduled for about 90 minutes | Qualitative data collection procedure, 15, 16. |
| 15. Presence of non-participants | *Was anyone else present besides the participants and researchers?*  No, only the interviewer, the observer and the participant were in the space during the interview. | Qualitative data collection procedure, 15,16. |
| 16. Description of sample | *What are the important characteristics of the sample? e.g. demographic data, date*  Participated in the study 115 HCPs, whom worked daily with P. vivax malaria diagnosis, treatment and health education or oversaw a facility treating malaria patients, had received the training, and consented to participation. | Sampling, 9.  Participant characteristics, 17, 18. |
| *Data collection* |  |  |
| 17. Interview guide | *Were questions, prompts, guides provided by the authors? Was it pilot tested?*  The questions were developed by the researchers with experience in malaria and qualitative research. | Qualitative data collection procedure, 15, 16. |
| 18. Repeat interviews | *Were repeat inter views carried out? If yes, how many?*  No | Qualitative data collection procedure, 15, 16. |
| 19. Audio/visual recording | *Did the research use audio or visual recording to collect the data?*  The interviews were recorded and transcribed without personal identifiers, so that the database could be anonymized | Qualitative data collection procedure, 15, 16. |
| 20. Field notes | *Were ﬁeld notes made during and/or after the inter view or focus group?*  Field notes were recorded by the interviewer and also by the observer. | Qualitative data collection procedure, 15, 16. |
| 21. Duration | *What was the duration of the interviews or focus group?*  The IDIs duration was 35 minutes, while FGs about 90 minutes. | Qualitative data collection procedure, 15, 16. |
| 22. Data saturation | *Was data saturation discussed?*  The number of interviews was determined by the principle of theoretical saturation where SIs are carried out until a clear pattern appears and subsequent groups do not produce new information | Sampling, 9. |
| 23. Transcripts returned | *Were transcripts returned to participants for comment and/or correction?*  No. | N/A |
| **Domain 3: analysis and ﬁndings** |  |  |
| *Data analysis* |  |  |
| 24. Number of data coders | *How many data coders coded the data?*  Two researchers (FM and AP) collaborated to build a consensus on the categories. Additionally, three researchers (MF, AP, and FR) developed a code book and performed line-by-line coding. | Data analysis,16, 17. |
| 25. Description of the coding tree | *Did authors provide a description of the coding tree?*  No. | N/A |
| 26. Derivation of themes | *Were themes identiﬁed in advance or* *derived from the data?*  The analysis of the interviews and the field notes allowed us to identify the four major themes. | Qualitative data collection procedure, 15, 16. |
| 27. Software | *What software, if applicable, was used to manage the data?*  The recordings of the IDIs and FDGs were transcribed and inserted in the MAXQDA 20 program | Data analysis, 16, 17. |
| 28. Participant checking | *Did participants provide feedback on the ﬁndings?*  No. | N/A |
| *Reporting* |  |  |
| 29. Quotations presented | *Were participant quotations presented to illustrate the themes/ﬁndings? Was each quotation identiﬁed? e.g. participant number*  Yes, quotations were presented to illustrate the themes/findings, and each quotation was identified with a participant number. | Results, 17-31. |
| 30. Data and ﬁndings consistent | *Was there consistency between the data presented and the ﬁndings?*  Yes, there was consistency between the data presented and the findings. | Discussion, 33-41. |
| 31. Clarity of major themes | *Were major themes clearly presented in the ﬁndings?*  Yes, major themes were clearly presented in the Results section using specific sections regarding each theme. | Results, 17-31. |
| 32. Clarity of minor themes | *Is there a description of diverse cases or discussion of minor themes?*  No, minor themes were not discussed. | N/A |
