# Supplementary material for: Perspectives of healthcare professionals on training for quantitative G6PD testing during implementation of tafenoquine in Brazil (QualiTRuST Study)
Source: PLoS Negl Trop Dis. 2024 Jun 5;18(6):e0012197. doi: 10.1371/journal.pntd.0012197 (PMC11152287; doi:10.1371/journal.pntd.0012197)
Supplement: S2 File — (DOCX) [file pntd.0012197.s002.docx]

**S2 File-Test for physicians who completed the online course**

- With the help of the following figure, answer the next 2 questions:


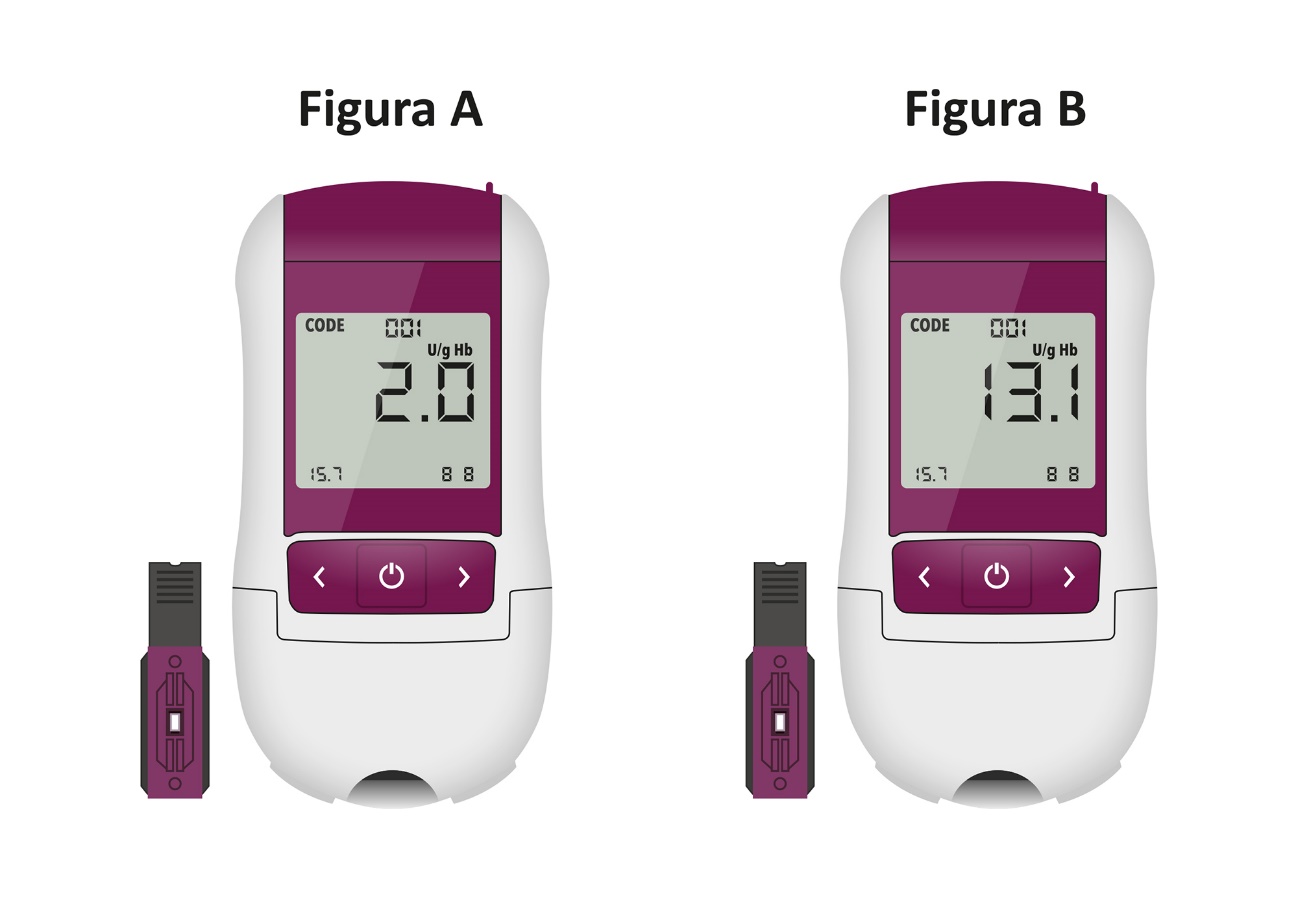


1. According to **Figure A**, what is the best treatment recommended for a patient with vivax malaria?
   1. Chloroquine - 3 days and Primaquine - 7 days.
   2. Chloroquine - 3 days and Weekly chloroquine for 12 weeks.
   3. **Chloroquine - 3 days and Weekly primaquine for 8 weeks.**
2. According to **Figure B**, what is the best treatment recommended for a patient with vivax malaria?
   1. **Chloroquine - 3 days and Single-dose Tafenoquine.**
   2. Chloroquine - 3 days and Weekly Tafenoquine for 12 weeks.
   3. Chloroquine - 3 days and Weekly Primaquine for 8 weeks.
3. If a patient has a G6PD test result of 1.8, he:
   1. **Cannot use Primaquine for 7 days and will use Primaquine for 8 weeks.**
   2. Can use Primaquine for 7 days.
   3. Will use Tafenoquine.
4. Which option shows what should be informed to the patient after getting the G6PD test and receiving treatment?
   1. **Return to do the LVC ( Malaria thick smear cure verification) on the fifth day of treatment.**
   2. All options are correct.
   3. Be aware of signs and symptoms of hemolysis such as dark-colored urine, yellow eyes, and dizziness.
