# Supplementary material for: Perspectives of healthcare professionals on training for quantitative G6PD testing during implementation of tafenoquine in Brazil (QualiTRuST Study)
Source: PLoS Negl Trop Dis. 2024 Jun 5;18(6):e0012197. doi: 10.1371/journal.pntd.0012197 (PMC11152287; doi:10.1371/journal.pntd.0012197)
Supplement: S3 File — (DOCX) [file pntd.0012197.s003.docx]

**S3-** **Lesson Plan for Training in the Context of Tafenoquine Implementation and Quantitative G6PD Testing**

| **LESSON PLAN** | |
| --- | --- |
|  |  |
| TRAINING FOR IMPLEMENTATION OF G6PD TEST/TAFENOQUINE | |
| DATE: XX/XX/2022 | TIME: XXX |
| LOCATION: CEMETRON/CEPEM LABORATORY AND DOCTOR HEITOR VIEIRA DOURADO HOSPITAL (FMT-HVD) | |

| ESTIMATED DURATION | CONTENTS | OBJECTIVE | METHODOLOGY | TEACHING RESOURCES |  |
| --- | --- | --- | --- | --- | --- |
| 25 minutes  Theory | Unit 1 - implementation and TRuST study | Capture the level of knowledge of technicians about the changes in the service protocol via debates. Explain about the question box activity in the training.  Changes in the treatment of malaria; to signify the effort and engage the professional in new practices, expose that the change is a guideline from the Ministry of Health;  Emphasize the difference between implementation and TRuST; to avoid sharing of incorrect information among professionals;  Importance of the implementation; to engage the professional in training;  Importance of the observation carried out by the TRuST study; to engage the professional in the collection and active search for data;  Consent; to clarify the way in which this should be approached and avoid the sharing of incorrect information;  Concept of and need for G6PD testing;  Note: Avoid the use of the word study so that professionals do not reproduce it during visits | Dialogued class  Practice (dramatization): ask professionals to reproduce a consent request from a standard text to verify that they have understood the goal | Projector  Slides  Microphone  10 slides |  |
| Break  15 min | Interval | | | |  |
| 25 minutes | Unit 2 -  Treatment | Presentation of the G6PD test for professionals; explain about G6PD deficiency and give a brief introduction about its relationship with hemolytic anemia.  Point out the advantages of TQ treatment; explore benefits, advances and emphasize its use as a primary method of treatment;  Composition of the drug, emphasize which elements are present and avoid confusion of professionals regarding allergies and intolerance, highlight that there are no relief drugs and explore drug interaction;  Describe the main adverse events associated with the drug (TQ), show that they are similar to those of PQ;  Difference between TQ and PQ, explain the specificities in their prescription and use, and the effects of TQ compared to PQ; emphasizing the benefits of TQ;  Highlight that cases of hemolysis can occur with both PQ and TQ;  TQ does not replace PQ, TQ will become the priority treatment for those patients eligible to take it; however, PQ remains a cure option for others.  Highlight that TQ requires attention to certain criteria for its administration, to sensitize them and deceive them in complying with all the stages of the new treatment guidelines;  Decision algorithms for treatment; elucidate the factors and possible strategies related to treatment. Highlight a flow of care according to the result of the G6PD test; specific groups for emphasizing mainly the treatment for infants and pregnant women; | Dialogued class  True or false | Projector  Slides  Microphone |  |
| 20 minutes  Theory | Adverse events | Dark urine, demonstrate what dark urine is and differentiate it between common colors of urine;  Warning signs for identifying hemolysis, highlight the signs and their significance and risk. | Dialogued class | Present urine in transparent pots for better fixation |  |
| 15 minutes  Practice | Activity: request that the questions be written on a small piece of paper and deposited in a box, then the class mediator removes the slips of paper and talks about their doubts. Some questions can be answered by the participants themselves. | | | |  |
| 25 minutes  Theory | SIVEP  and  CCS | Importance of correctly filling in the SIVEP form;  Most significant examples of the result such as 00.9; emphasize the presence of the comma;  Use less technical terms during training;  Malaria card - stress its importance.  Definition of the CCS  When to test G6PD deficiency on a positive CCS  How long after the first malaria episode should the CCS be done?  Correct filling in of the SIVEP form for negative and positive CCS | Dialogued class with problem solving | Projector  Slides  Microphone |  |
| 20 minutes  Practice | Activity: clinical cases for defining treatment. The objective of this activity is to explore the importance of the G6PD test in order to define the treatment for both tafenoquine and primaquine. Present problem cases in order to verify the need to redo the test. The purpose of this activity is to emphasize the need to retest in women and when it is not necessary to redo the test. | | | |  |
| 20 minutes | Activity: copies of SIVEP DEMO 3 will be distributed to health care professionals and they will be asked to fill the form in with the data of a fictitious patient. The activity will be corrected by the participants themselves and, at the end, they will be presented with feedback. The objective of this activity is to emphasize the importance of consent/authorization and the best way to explain this aspect to the patient, without the use of terms that may confuse them. | | | |  |
| Total estimated time: 240 minutes (4 hours) | | | | | |
| REFERENCES | | | | | |
|  | 1. MARQUES, Humberto Rodrigues et al. Inovação no ensino: uma revisão sistemática das metodologias ativas de ensino-aprendizagem. Avaliação: Revista da Avaliação da Educação Superior (Campinas) [online]. 2021, v. 26, n. 03 [Acessado 20 dezembro 2021], pp. 718-741. Disponível em: <https://doi.org/10.1590/S1414-40772021000300005>. Epub 10 Dez 2021. ISSN 1982-5765. <https://doi.org/10.1590/S1414-40772021000300005>. 2. COIMBRA, Camila Lima. A aula expositiva dialogada em uma perspectiva freireana. *LEAL, Edvalda Araújo; MIRANDA, Gilberto José; CASA NOVA, Silvia Pereira de Castro. Revolucionando a Sala de Aula: como envolver o estudante aplicando técnicas de metodologias ativas de aprendizagem. São Paulo: Atlas* (2017): 1-13. 3. BRAUER, Markus. Ensinar na Universidade. Conceitos Práticos, Dicas, Métodos Pedagógicos. Volume I Capa comum – Parábola ed, 1 - 8 outubro 2012. | | | |  |
|  | The QualiTRuST team suggests that active methodologies be used to engage and stimulate the professionals present in the training. The dialogued expository lesson method offers the opportunity to teach the relevant theoretical content and articulates it with the professional experience of the participants, so that it facilitates understanding and fixation. This method creates an environment for sharing experiences and particular issues related to professional practice, which stimulates them to clarify possible doubts without feeling inhibited. | | | |  |
