# Supplementary material for: Perspectives of healthcare professionals on training for quantitative G6PD testing during implementation of tafenoquine in Brazil (QualiTRuST Study)
Source: PLoS Negl Trop Dis. 2024 Jun 5;18(6):e0012197. doi: 10.1371/journal.pntd.0012197 (PMC11152287; doi:10.1371/journal.pntd.0012197)
Supplement: S5 File — (DOCX) [file pntd.0012197.s005.docx]

**S5 File- Cards designed for distribution via WhatsApp about the main mistakes and doubts that arose after the training sessions.**

**CARD 1**


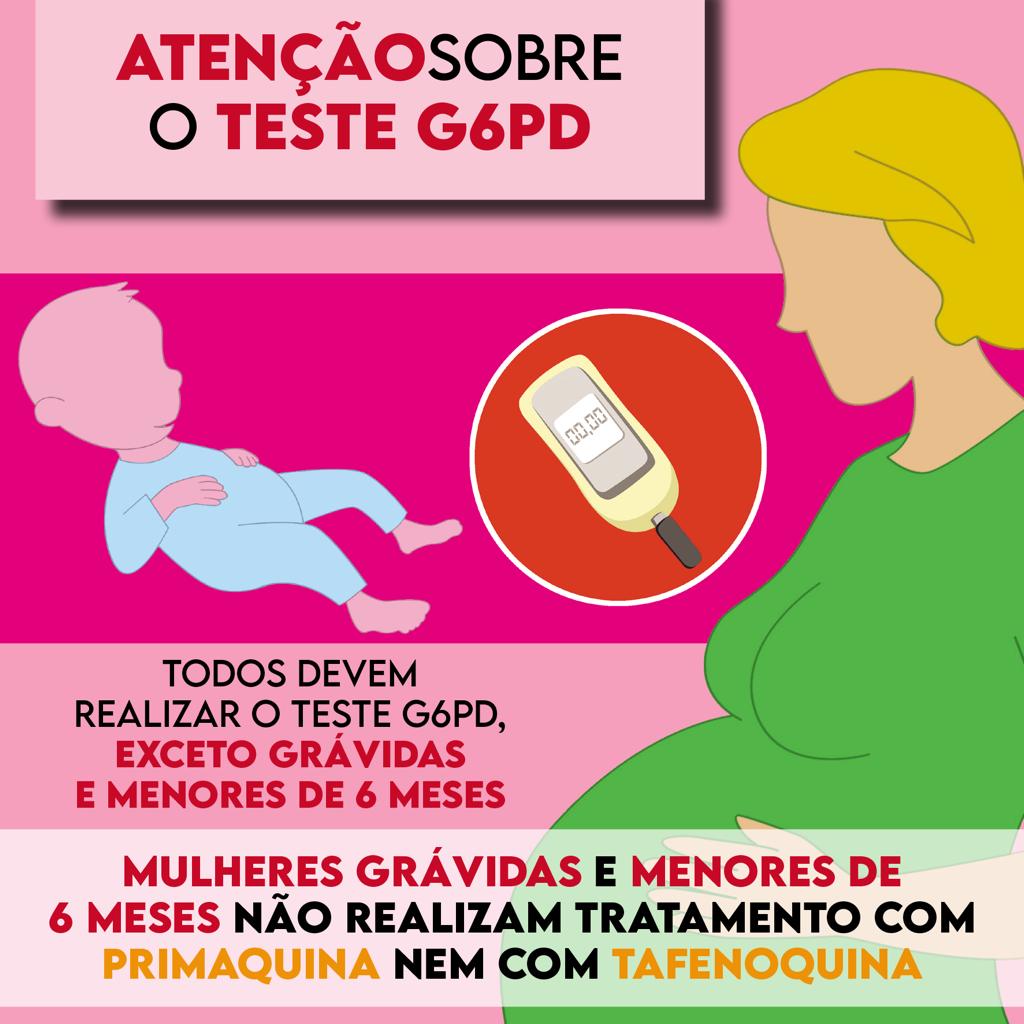


**Text in English:**

**ATTENTION ABOUT** **THE G6PD TEST**

**EVERYONE SHOULD TAKE THE G6PD TEST,** **EXCEPT PREGNANT WOMEN AND UNDER 6**

**PREGNANT WOMEN AND CHILDREN UNDER 6 MONTHS OF AGE DO NOT UNDERGO TREATMENT WITH PRIMAQUINE OR TAFENOQUINE.**

**CARD 2**


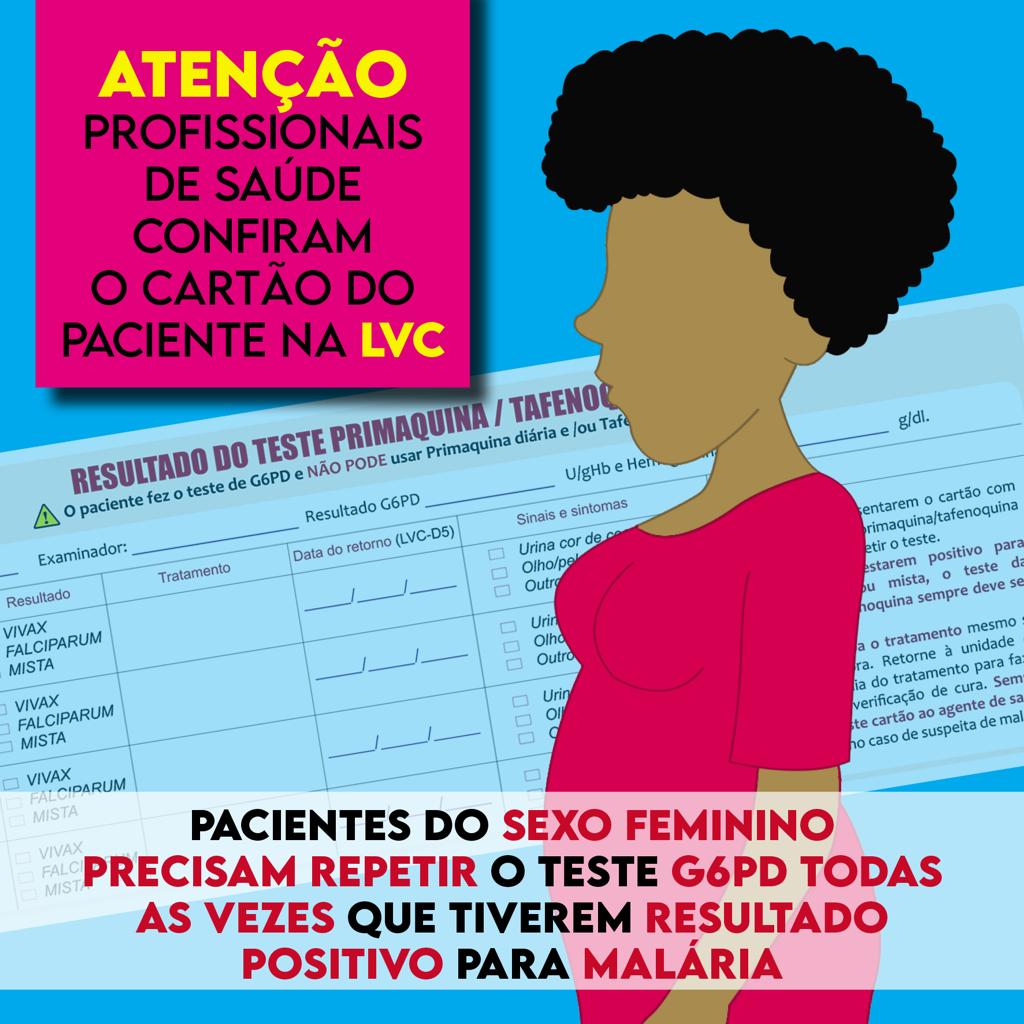


**Text in English:**

**ATTENTION HEALTH PROFESSIONALS CHECK THE PATIENT'S CARD UPON RETURN**

**FEMALE PATIENTS NEED TO REPEAT THE G6PD TEST EVERY TIME THEY HAVE A POSITIVE RESULT FOR MALARIA**

**CARD 3**


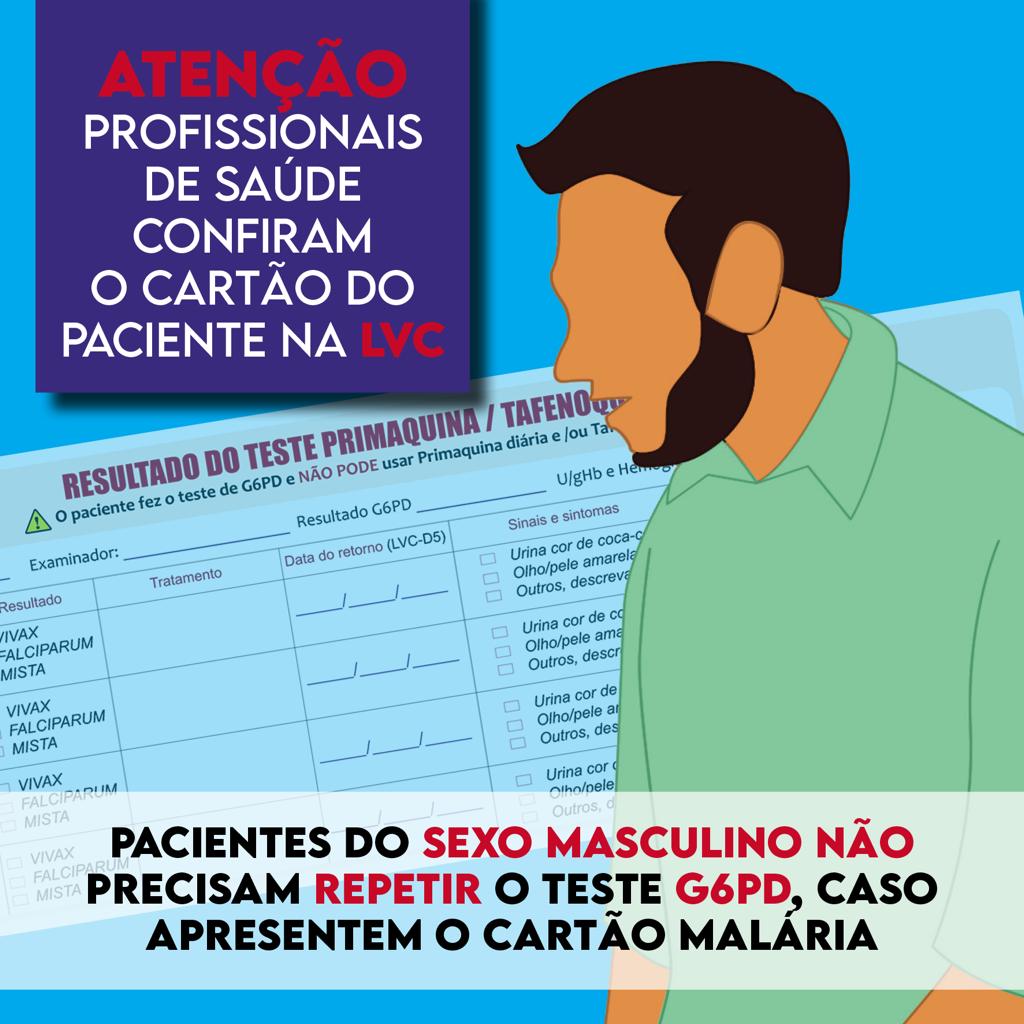


**Text in English:**

**ATTENTION HEALTH PROFESSIONALS CHECK THE PATIENT'S CARD UPON RETURN**

**MALE PATIENTS DO NOT NEED TO REPEAT THE G6PD TEST IF THEY PRESENT THE MALARIA CARD**
